# Front-of-pack nutrition labelling: time for the EU to adopt a harmonized scheme

**DOI:** 10.1093/eurpub/ckad087

**Published:** 2023-06-09

**Authors:** Nikhil Gokani, Amandine Garde

**Affiliations:** Law and Public Health Section, European Public Health Association; School of Law, University of Essex, Essex, UK; Law and Public Health Section, European Public Health Association; Law & Non-Communicable Diseases Research Unit, School of Law and Social Justice, University of Liverpool, Liverpool, UK

In its Farm to Fork Strategy, published nearly 3 years ago in May 2020, the European Commission committed to ‘propose harmonised mandatory front-of-pack nutrition labelling’ (‘FoPNL’) to ‘empower consumers to make informed, healthy and sustainable food choices’ by the fourth quarter of 2022. This commitment was repeated in Europe’s Beating Cancer Plan in February 2021. The deadline has now passed and the promised proposals do not seem forthcoming. This is all the more disappointing considering there is strong support for the implementation of an EU-wide harmonized FoPNL scheme, as demonstrated by the results of the EU consultation on ‘Food labelling—revision of rules on information provided to consumers’ published in December 2021.

Such support is not surprising considering the significant advantages that the adoption of a harmonised FoPNL scheme has for consumers, traders, Member States and the EU alike.^1^

- From the perspective of consumers, an effectively designed FoPNL scheme helps inform them of the nutritional composition of food. Informing consumers lies at the heart of the EU’s consumer protection strategies and reflects its long-held view that regulating food labelling empowers consumers to make healthier choices whilst promoting the objectives of market integration.^2^ At present, the EU only mandates a small table of nutrition information on the back of food packaging. This is often hard to see and difficult to understand, whereas effectively designed FoPNL can provide easy-to-see and easy-to-understand information on the front of food packaging thus supporting healthier food choices.^3^
- From the perspective of traders, harmonized FoPNL will create a level playing field by reducing regulatory fragmentation, which will also increase legal certainty and lower labelling costs. There are currently 7 national FoPNL schemes recommended across 15 EU Member States. Further industry-led schemes are used, although they have not been officially endorsed by any Member State. While some manufacturers have adopted FoPNL, many have not, and others are using multiple different schemes.
- From the perspective of Member States, a mandatory, EU-wide FoPNL scheme will contribute to improving diets and health outcomes. Current EU rules prohibit the adoption of FoPNL schemes which are interpretive, and do not facilitate the adoption of FoPNL schemes which are easy to use. They also prevent Member States from making FoPNL mandatory.^4^
- From the perspective of the EU itself, a harmonized FoPNL scheme will promote the proper functioning of the internal market in line with the EU’s mandate to ensure a high level of health and consumer protection in all its policies. Moreover, it will facilitate the compliance of all its Member States with the commitments that they have made at international level to promote healthier food environments.^5^

The choice of any single scheme must be guided by evidence. The Commission’s Joint Research Centre reviews, published in 2020 and 2022, identify what makes FoPNL effective:

- colour-coded labels draw consumer attention through increased salience, are preferred by consumers, are associated with increased understanding and encourage healthier food purchases;
- simple labels require less attention to process and are preferred and more easily understood by consumers; and
- consumers prefer and better understand consistent and simple reference quantities.

In its Inception Impact Assessment of December 2020, the Commission put forward four types of labels as contenders for a harmonized, mandatory EU-wide scheme: graded indicators (e.g. Nutri-Score); endorsement logos (e.g. Keyhole); colour-coded (e.g. Multiple Traffic Lights); and numerical (e.g. NutrInform). It is clear that of the four schemes considered in the Inception Impact Assessment, Nutri-Score is the only one meeting the criteria above, and its effectiveness is strongly established. Not only does it attract consumers’ attention, it is favourably perceived and well understood. It also has a positive impact on the nutritional quality of purchases. Additionally, the nutrient profiling model underpinning Nutri-Score has been extensively validated and shown to be associated with improved health outcomes.^6^ Even if no scheme will ever be described as ‘perfect’ by all stakeholders, its developed evidence base and adoption by a growing number of Member States, makes Nutri-Score the only viable option for the timely implementation of a mandatory, harmonised FoPNL scheme in the EU.

Growing rates of obesity and other diet-related diseases increase the urgency for the EU to act. We therefore call on the Commission to propose legislation requiring food to be labelled with Nutri-Score on a mandatory basis across the EU, as it has committed to do.

## Data Availability

No new data were generated writing this editorial.
